# Simultaneous Inhibitory Effects of All-Trans Astaxanthin on Acetylcholinesterase and Oxidative Stress

**DOI:** 10.3390/md20040247

**Published:** 2022-03-31

**Authors:** Xin Wang, Tao Zhang, Xiaochen Chen, Yating Xu, Zhipeng Li, Yuanfan Yang, Xiping Du, Zedong Jiang, Hui Ni

**Affiliations:** 1 College of Food and Biological Engineering, Jimei University, Xiamen 361021, China; xinwang@jmu.edu.cn (X.W.); 201911710026@jmu.edu.cn (T.Z.); 202011832012@jmu.edu.cn (X.C.); xuyating@jmu.edu.cn (Y.X.); yuanfan@jmu.edu.cn (Y.Y.); zdjiang@jmu.edu.cn (Z.J.); nihui@jmu.edu.cn (H.N.); 2 Fujian Provincial Key Laboratory of Food Microbiology and Enzyme Engineering, Xiamen 361021, China; 3 Research Center of Food Biotechnology, Xiamen 361021, China; 4 Key Laboratory of Systemic Utilization and In-Depth Processing of Economic Seaweed, Xiamen Southern Ocean Technology Center of China, Xiamen 361021, China; 5 Collaborative Innovation Center of Seafood Deep Processing, Dalian Polytechnic University, Dalian 116034, China

**Keywords:** Alzheimer´s disease, acetylcholinesterase, all-trans astaxanthin, oxidative stress

## Abstract

Alzheimer´s disease is a global neurodegenerative health concern. To prevent the disease, the simultaneous inhibition of acetylcholinesterase and oxidative stress is an efficient approach. In this study, the inhibition effect of all-trans astaxanthin mainly from marine organisms on acetylcholinesterase and oxidative stress was evaluated by a chemical-based method in vitro and cell assay model. The results show that all-trans astaxanthin was a reversible competitive inhibitor and exhibited a strong inhibition effect with half inhibitory concentration (IC_50_ value) of 8.64 μmol/L. Furthermore, all-trans astaxanthin inhibited oxidative stress through reducing malondialdehyde content and increasing the activity of superoxide dismutase as well as catalase. All-trans astaxanthin could induce the changes of the secondary structure to reduce acetylcholinesterase activity. Molecular-docking analysis reveals that all-trans astaxanthin prevented substrate from binding to acetylcholinesterase by occupying the space of the active pocket to cause the inhibition. Our finding suggests that all-trans astaxanthin might be a nutraceutical supplement for Alzheimer´s disease prevention.

## 1. Introduction

Alzheimer’s disease (AD), the most common form of dementia, is a progressive neurodegenerative syndrome [1]. These disorders have a high prevalence and short-/long-term impairments and disabilities. Therefore, there are emotional, financial, and social burdens to the patients and their families [2]. Around 50 million people worldwide have this disease, and nearly 10 million new cases occur every year. Direct and indirect medical expenditure that is related to AD and other dementias is estimated to around $148 billion and that value is rising annually [3]. 

One of the remarkable biochemical changes in AD patients is a reduction of the acetylcholine levels in the hippocampus and cortex of brain [4]. The level of acetylcholine is regulated by acetylcholinesterase which is the key enzyme in the breakdown and hydrolysis of acetylcholine [5]. Sufficient acetylcholine is required for proper brain functioning. AD patients suffer from progressive decline in cognitive functioning and behavioral abilities owing to acetylcholine decrease [6]. Therefore, the inhibition of acetylcholinesterase has been proved to help in slowing down the disease and considered as a strategy for the prevention [7]. 

On the other hand, oxidative stress is also pathologically connected with cognitive deficits that occur during AD [8]. This stress might be a consequence of the tissue injury, in a vicious cycle of actions and reactions resulting in a critical mass of metabolic errors that are responsible for this disease in the end [9]. More specifically, oxidative stress in brain tissue gradually kills neurons in the cortex, hippocampus, and other regions [10]. Medical efforts that are aimed at removal of oxidative stress or prevention of their formation may be beneficial in AD [11].

The use of acetylcholinesterase inhibitors is widely approved in the treatment and prevention of Alzheimer’s disease [12]. Several inhibitors aiming at the prevention of acetylcholine degradation have been designed and synthesized [13]. Galantamine benefits the cognitive, functional, and behavioral symptoms for AD management [14]. However, it also has side effects including central nervous system events, extrapyramidal symptoms, and sleep disturbances events. Thus, developing effective antioxidant inhibitors with minimal side effects offers an attractive strategy for managing AD.

All-trans astaxanthin is a natural carotenoid with both antioxidant and neuroprotective effects [15], mainly from marine organisms. On one hand, astaxanthin in nerve cells protects the mitochondria against endogenous oxygen radicals, conserves their antioxidant capacity, and enhances energy production efficiency [16]. On the other hand, unreasonable intracellular levels of lipid peroxide might cause damage to lipids and proteins [17], thus malondialdehyde as potential biomarker is one of the most frequently used indicators of lipid peroxidation [18]. Additionally, clearance of hydrogen peroxide and superoxide contributes to reducing cellular injury under the stress response, therefore the corresponding antioxidant enzyme activities such as catalase and superoxide dismutase are evaluation indicators. Thus, astaxanthin would be a candidate for the prevention of AD.

In previous studies anti-oxidative stress effects of astaxanthin have been reported by using different evaluation models. However, the information regarding astaxanthin upon lipid peroxidation production and antioxidant enzymes in the AD cell model remains unknown. Also, the inhibitory effect of astaxanthin on acetylcholinesterase is still not clear. Therefore, the simultaneous inhibitory effect of all-trans astaxanthin on antioxidant enzymes, acetylcholinesterase, and lipid peroxidation production was evaluated in this study. Firstly, a chemical-based method, PC12 and modified PC12 cells assay in vitro were used to detect the enzyme activity and cell viability, respectively. Furthermore, fluorescence spectroscopy and circular dichroism spectroscopy were used to investigate the conformation change of acetylcholinesterase. Last, molecular docking analysis was used to reveal the inhibition and binding mechanism. The findings will provide useful information for development of more effective anti-dementia agents and provide scientific evidence for extending the application of astaxanthin in nutraceutical industries.

## 2. Results and Discussion

### 2.1. Effect of All-Trans Astaxanthin on Acetylcholinesterase Activity In Vitro

**Figure 1:**
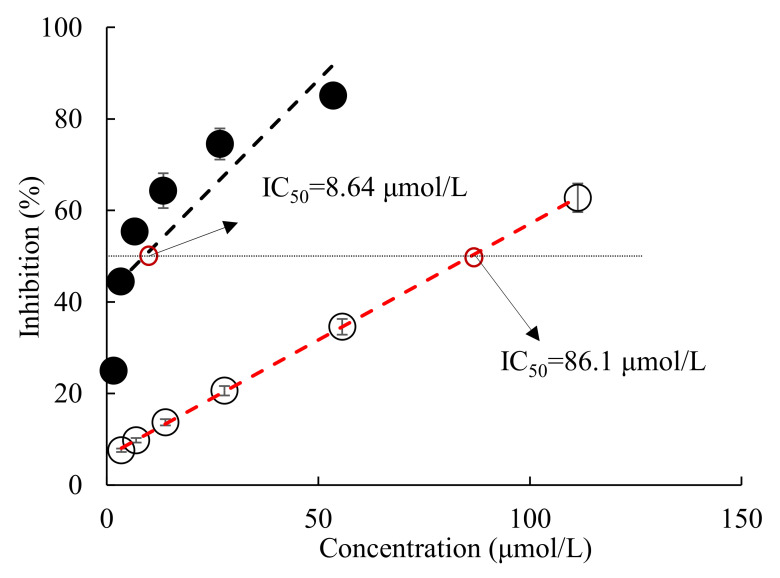
Inhibition of acetylcholinesterase by all-trans astaxanthin and galantamine. ●: all-trans astaxanthin; ○: galantamine.

Figure 1 shows that all-trans astaxanthin inhibited acetylcholinesterase in a dose-dependent manner with IC_50_ value of 8.64 μmol/L, whereas that value of positive control galantamine was 86.1 μmol/L. Obviously, acetylcholinesterase was much more sensitive to the inhibition by all-trans astaxanthin than by galantamine.

All-trans astaxanthin is a potent natural antioxidant and has important applications in the nutraceutical, cosmetics, food, and feed industries [19]. It also has other beneficial bioactivities to human health including scavenging free radical bioactivity [20], anti-inflammatory [21], and hypoglycemic effects [22]. In previous studies, astaxanthin with a 700–920 mg/kg bw/day carbohydrate formulation do not exhibit adverse effects in safety assessment [23]. Therefore, all-trans astaxanthin might be an efficient and safe acetylcholinesterase inhibitor.

### 2.2. Enzyme Kinetic Study

**Figure 2:**
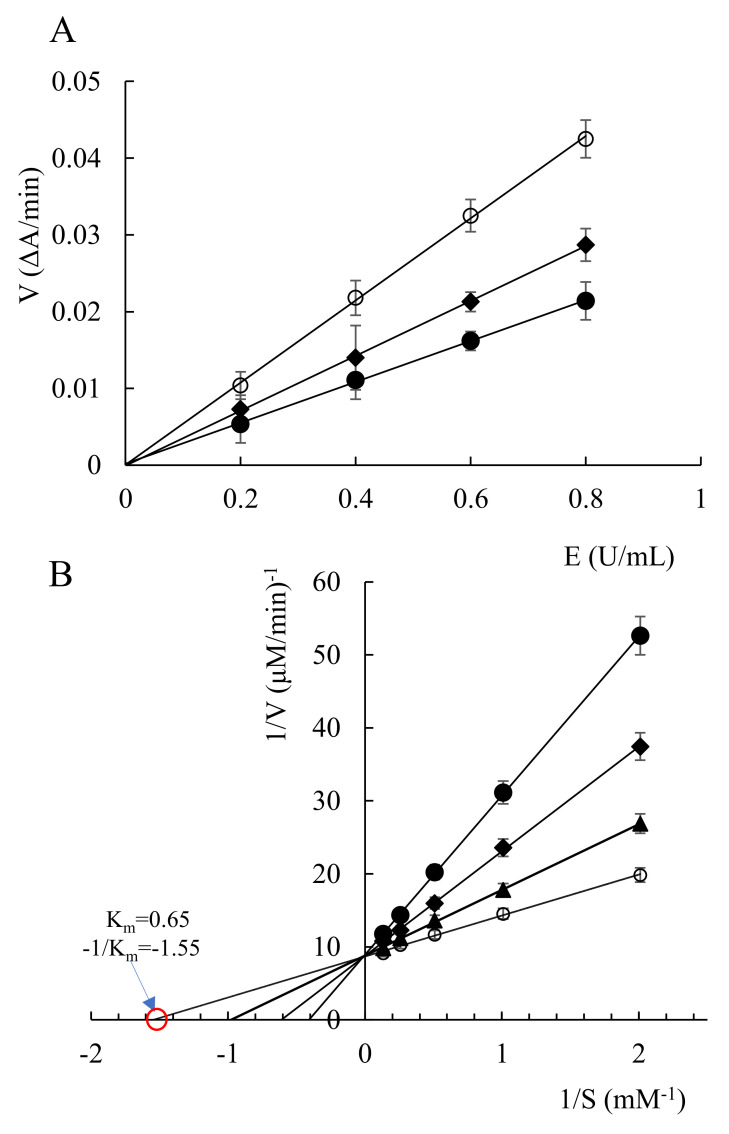
Reaction rates of acetylcholinesterase in the presence of all-trans astaxanthin with different concentrations (**A**), and Lineweaver–Burk reciprocal plots (**B**). ○: all-trans astaxanthin concentration 0 μmol/L; ▲: all-trans astaxanthin concentration 6.5 μmol/L; ◆: all-trans astaxanthin concentration 26 μmol/L; ●: all-trans astaxanthin concentration 52 μmol/L.

Figure 2A shows that a linear relationship could be observed between acetylcholinesterase concentrations and the reaction speed. With a concentration increase, the reaction speed gradually increased in the measured range. All the curves passed through the origin point, whereas the slope decreased along with the increase of the all-trans astaxanthin concentration. That means that all-trans astaxanthin bound to acetylcholinesterase by non-covalent bonds, resulting in the decrease or loss of enzymatic activity, whereas the enzyme can be reactivated by the physical removal of the inhibitors [24]. These results indicate that the inhibition was reversible.

Figure 2B shows the relationship between substrate concentrations and reaction speed. Based on the obtained Lineweaver–Burk plots, a competitive-type inhibition pattern for all-trans astaxanthin could be assigned as the lines intersected in the *y* axis. According to the Michaelis–Menten equation, the value of the intersection of the curve (all-trans astaxanthin concentration was 0 μmol/L) and the *x* axis was calculated as −1/Km value (−1.55 μmol/L). Thus, the Km value was 0.65 μmol/L. The results show that all-trans astaxanthin could inhibit the catalysis activity of acetylcholinesterase via competing with the binding active site of acetylcholinesterase with the substrate.

### 2.3. Changes in Acetylcholinesterase Conformation Based on Circular Dichroism Spectra and Fluorescence

**Figure 3:**
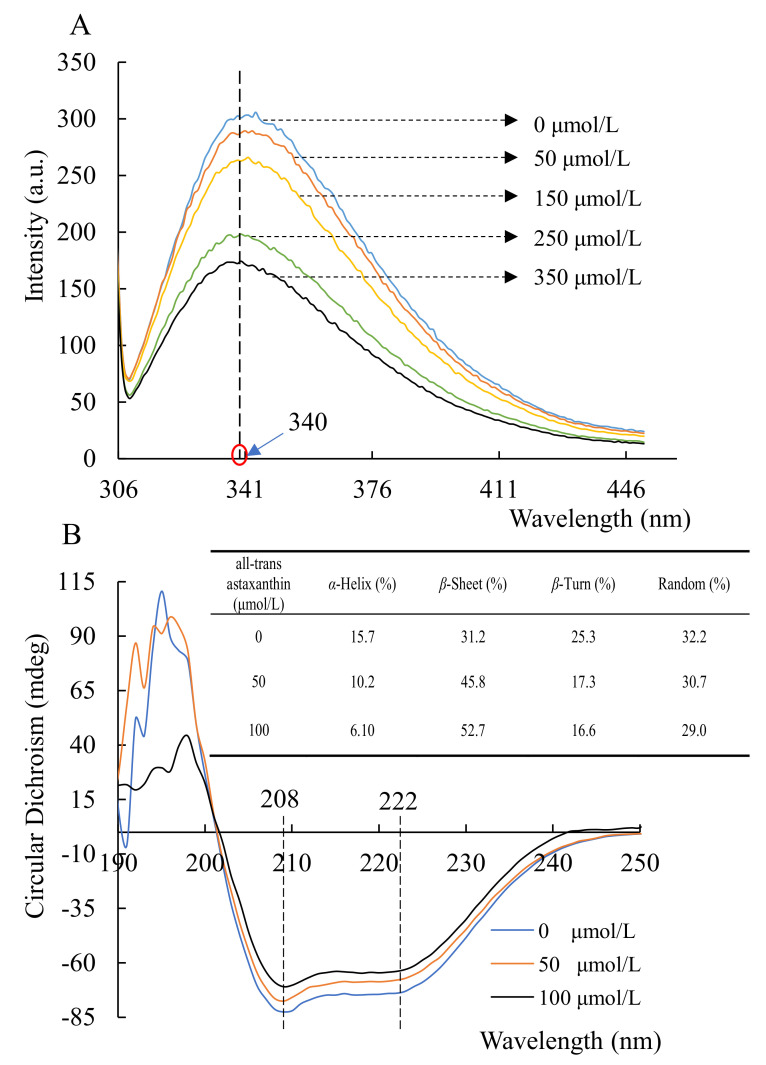
Fluorescence emission (**A**) and circular dichroism (**B**) spectra of acetylcholinesterase in the presence of all-trans astaxanthin with various concentrations.

Figure 3A shows that acetylcholinesterase had a fluorescence intensity at 340 nm wavelength. In the enzyme-inhibitor system where the all-trans astaxanthin concentration increased, the recorded fluorescence intensity of the mixture decreased progressively. That means all-trans astaxanthin interacted with acetylcholinesterase as an inhibitor. A previous study reports that fluorescence quenching is attributed to electrostatic interaction [25]. Therefore, all-trans astaxanthin might produce intermolecular energy when combined with acetylcholinesterase. The hydrophobic or hydrogen bonds were formatted between acetylcholinesterase and all-trans astaxanthin.

Figure 3B shows that two negative peaks were observed at 208 and 222 nm. They are the main characteristics of α-helix and β-sheet of acetylcholinesterase, respectively [26]. The intensity of α-helix and β-sheet changed when all-trans astaxanthin was added in the mixture, indicating that all-trans astaxanthin might induce the change of secondary structure in acetylcholinesterase. Figure 3B shows that the content of secondary structure changed along with the increased concentration of all-trans astaxanthin from 0 to 100 μmol/L. The content of α-helix decreased from 15.7 to 6.10%, of random coil decreased from 32.2 to 29.0%, and of β-turn decreased from 25.3 to 16.6%, whereas the β-sheet content increased from 31.2 to 52.7%. Thus, all-trans astaxanthin might destroy the hydrogen bonding networks of acetylcholinesterase molecules and induce the changes of the natural secondary structures.

### 2.4. Effect of All-Trans Astaxanthin Concentration on PC12 Cell Viability

**Figure 4:**
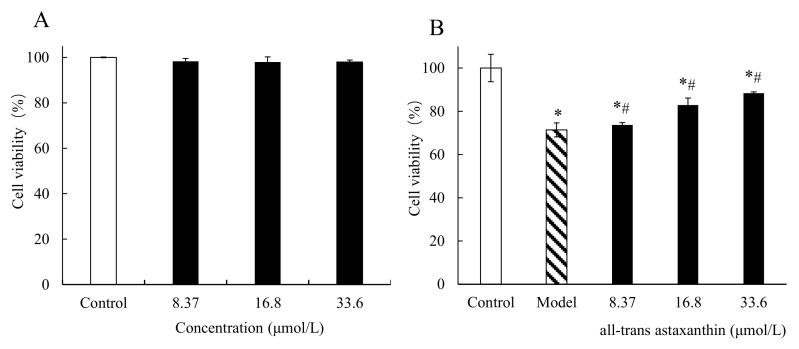
The effect of all-trans astaxanthin concentrations on cell viability in group (**A**) and in group (**B**) treated with Aβ25–35. □: Control group; ■: astaxanthin-treatment group; ▧: Model group; ∗ *p* < 0.05, compared with the control; # *p* < 0.05, compared with the model group.

Figure 4A shows the effect of all-trans astaxanthin concentrations on PC12 cell viability. The cell viability was 98.3% when concentration of all-trans astaxanthin was 8.37 μmol/L, and the value decreased to 98.0% when concentration of inhibitor increased to 33.6 μmol/L. Within this concentration range, no significant difference in cell viability was observed (*p* < 0.05). The results show that all-trans astaxanthin had no significant cytotoxicity at the measured doses (0 to 33.6 μmol/L) and could be used in the further assay in this study. 

Figure 4B shows the effect of all-trans astaxanthin concentrations on the viability of Aβ25–35-induced PC12 cells (AD model cells). In control group, PC12 cells grew quickly and adhered to the well wall. After treatment with Aβ25–35, cells were damaged with a cell number decrease and the cell viability significantly decreased to 71.4% (*p* < 0.05). The results shows that AD cell model was established after this treatment. On the contrary, all-trans astaxanthin significantly improved the cell viability along with concentration increase (*p* < 0.05) in astaxanthin-treatment group. When the concentration of all-trans astaxanthin was 8.37 μmol/L, cell viability was calculated as 73.7% while the value increased to 88.3% with inhibitor concentration of 33.6 μmol/L. Thus, the cell viability of the AD cell model increased after all-trans astaxanthin treatment.

### 2.5. Effect of All-Trans Astaxanthin on Intracellular Antioxidant Capacity and Acetylcholinesterase Activity

**Figure 5:**
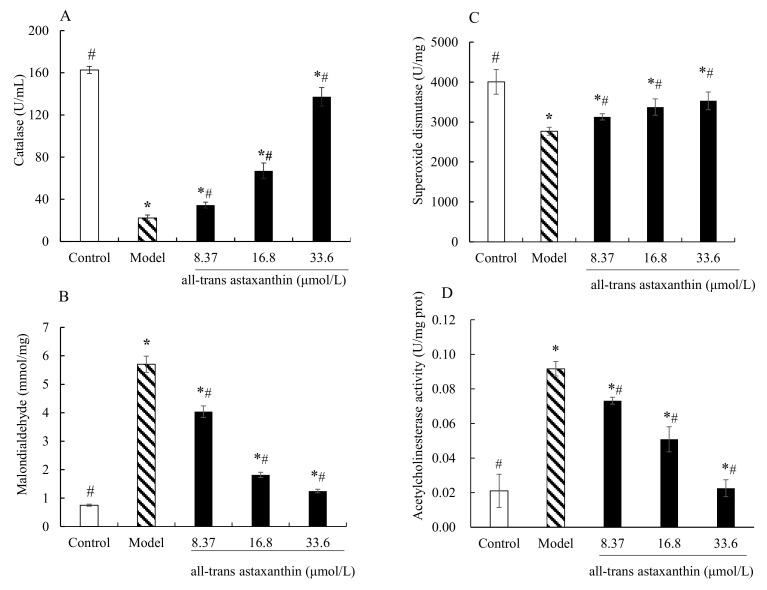
Effect of all-trans astaxanthin on intracellular antioxidant capacity and acetylcholinesterase activity. The levels of catalase (**A**), malondialdehyde (**B**), superoxide dismutase (**C**), and acetylcholinesterase activity (**D**) are shown. □: Control group; ■: Astaxanthin-treatment group; ▧: Model group; ∗ *p* < 0.05, compared with the control; # *p* < 0.05, compared with the model group.

Figure 5A shows the enzyme activity levels of catalase in different groups. The catalase activity of control group was 162 U/mL and that of AD group was 22.3 U/mL. In the astaxanthin-treatment group, the catalase activity significantly increased with concentration increase (*p* < 0.05) compared with model group. The catalase activity decreased to 137 U/mL with astaxanthin concentration of 33.6 μmol/L.

Figure 5B shows the contents of malondialdehyde in different groups. Compared with the control, the contents of malondialdehyde in AD model cells were significantly increased (*p* < 0.05). On the contrary, the levels of malondialdehyde decreased with all-trans astaxanthin concentration increase. The content was 4.03 mmol/mg at all-trans astaxanthin concentration of 8.37 μmol/L versus 1.24 mmol/mg at concentration of 33.6 μmol/L.

Figure 5C shows a similar trend in superoxide dismutase activity change. The superoxide activity in the control group was 4007.9 U/mg and that of AD group was 2768.6 U/mg. In the astaxanthin-treatment group, the superoxide dismutase activity significantly increased with a concentration increase (*p* < 0.05) compared with the model group. The enzyme activity was 3129.2 U/mg at the concentration of 8.37 μmol/L versus 3531.5 U/mg at all-trans astaxanthin concentration of 33.6 μmol/L.

Figure 5D shows the effect of astaxanthin concentrations on intracellular acetylcholinesterase activity in AD model cells. Acetylcholinesterase activity was 0.02 U/mg protein in the control group and the value significantly increased in AD model group of 0.09 U/mg protein. On the contrary, acetylcholinesterase activity decreased in the astaxanthin-treatment group with a concentration increase. The acetylcholinesterase activity was 0.02 U/mg protein with an all-trans astaxanthin concentration of 33.6 μmol/L. Therefore, all-trans astaxanthin exhibited an inhibitory effect on intracellular acetylcholinesterase activity in a dose-dependent manner.

Growing evidence has demonstrated that oxidative stress is an important factor contributing to the initiation and progression of AD [27]. PC12 cells that are derived from rat adrenal pheochromocytoma are commonly used in neuronal cell models [28]. Furthermore, Aβ25–35 peptide can induce oxidative stress both in vivo and in vitro [29]. Thus, in this study Aβ25–35 was used to induce PC12 cells and build an AD cell model. Then, changes in the content of malondialdehyde, as well as enzyme activity including catalase and superoxide dismutase were measured. The results show that when the concentration of Aβ25–35 was 20 μmol/L AD cell model was established. In model group, PC12 cell viability was decreased whereas malondialdehyde levels were significantly increased (*p* < 0.05). Meanwhile, the superoxide dismutase and catalase activity were significantly decreased (*p* < 0.05). That means Aβ25–35 induced an imbalanced oxidative system of the PC12 cells and produced oxidative stress. Compared with AD model, in astaxanthin-treatment group, the parameters including cell viability, catalase activity, and superoxide dismutase activity were significantly increased (*p* < 0.05). Meanwhile, the malondialdehyde levels significantly decreased (*p* < 0.05) after the treatment. 

Oxidative stress refers to elevated intracellular levels of lipid peroxide, that cause damage to lipids and proteins [17]. Malondialdehyde as a potential biomarker is one of the most frequently used indicators of lipid peroxidation [18]. The results show that astaxanthin-treatment reduced the production of end product of lipid peroxidation. Furthermore, the antioxidant defense system consists of enzymes such as catalase, superoxide dismutase and numerous non-enzymatic antioxidants [30]. Catalase catalyzes the conversion of hydrogen peroxide to water and molecular oxygen [31]. Superoxide dismutase breaks down superoxide molecules and prevents damage in cells [31]. Increased catalase activity and superoxide dismutase activity can enhance the cell’s decomposition efficiency of hydrogen peroxide. The results show that astaxanthin-treatment increased the catalase activity and superoxide dismutase activity compared with the AD model group. Thus, all-trans astaxanthin inhibited oxidative stress by reducing lipid peroxidation product content and improving the efficiency of hydrogen peroxide as well as superoxide clearance.

### 2.6. Molecular Docking Simulation of All-Trans Astaxanthin Binding to Acetylcholinesterase

**Figure 6:**
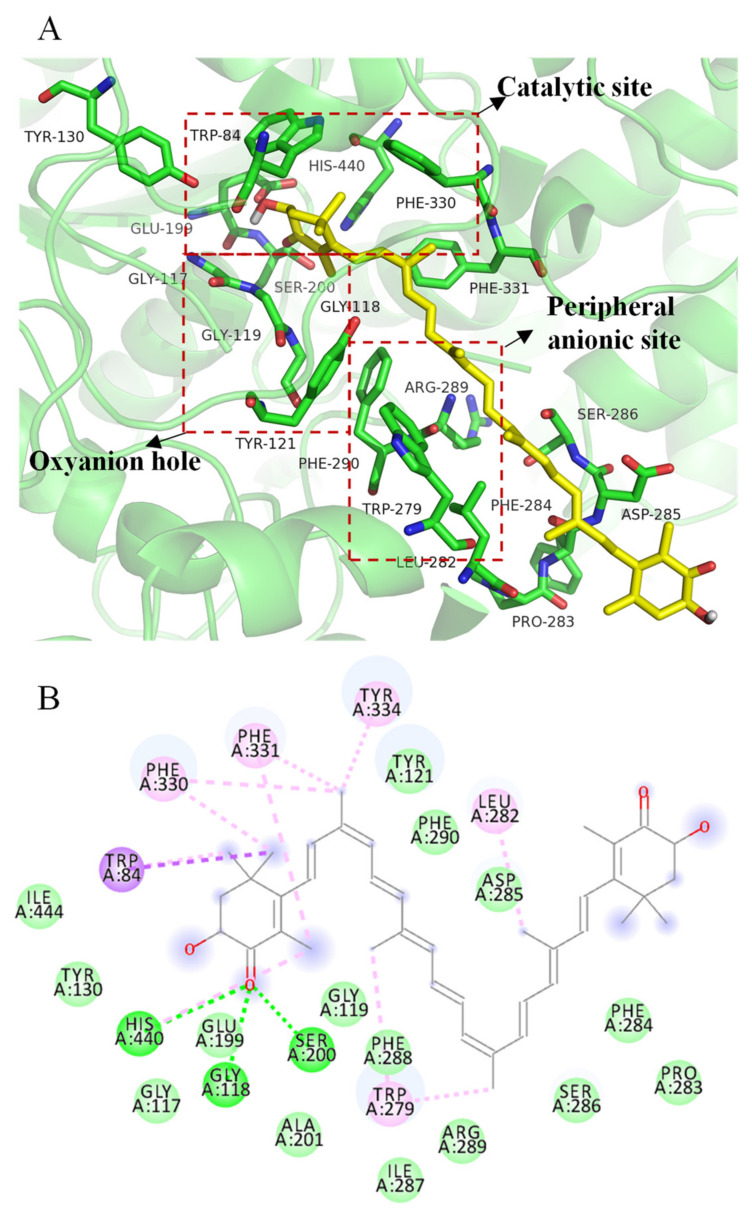
3D (**A**) and 2D (**B**) structural simulation of all-trans astaxanthin interacting with acetylcholinesterase.

Figure 6A shows that all-trans astaxanthin bonded to the key amino acids of major regions of acetylcholinesterase as a competitive inhibitor. The active center of acetylcholinesterase contains three major regions including the catalytic site, oxyanion hole, and peripheral anionic site [32]. The enzyme has a catalytic triad that is composed of Ser200, His440, and Glu327 at the bottom of the canyon, which catalyzes the acetyl hydrolysis of acetylcholine [33]. Trp84 and Phe330 are known as anionic subsites of the catalytic site that is involved in choline recognition through Carbon-π interaction [34]. A peripheral anionic site centered on Trp279 is located at the entrance of the active pocket and provides a binding site for allosteric modulators and inhibitors [35]. Gly118 and Gly119 form the oxyanion hole, which is located at the bottom of the active pocket and plays an important role in the enzymatic reaction [36]. Trp233, Phe288, Phe290, and Phe331 residues also form the acyl pocket involved in acetyl ester specificity [37]. Docking studies were carried out in our study to investigate the possible binding strategies of all-trans astaxanthin in the binding pocket and catalytic center.

Figure 6B shows that all-trans astaxanthin bonded to Ser200 and His440 in catalytic site by forming hydrogen bonds; bonded to Trp279 in peripheral anionic by forming the C-PI (alkyl) bond; bonded to Phe330 in anionic subsite by forming Carbon-π (alkyl) bond; and bonded to Trp84 in anionic subsite by forming Carbon-π (sigma) bond. 

The banding free energies of all-trans astaxanthin, galantamine, and substrate acetylthiocholine iodide were as follows. The value of all-trans astaxanthin was −10.7 kcal/mol, of galantamine was −9.7 kcal/mol, and of acetylthiocholine iodide was −4.4 kcal/mol. The low bonding energy indicates a stable binding strategy between the ligands (inhibitors or substrates) and target enzyme [38]. In comparison, the binding energy value of all-trans astaxanthin was the lowest among all the ligands, indicating that astaxanthin was a stable inhibitor candidate against acetylcholinesterase. In addition, the above kinetic analysis reveals that all-trans astaxanthin was a reversible competitive inhibitor. This type of inhibitor would compete with substrates to bind to the active center of an enzyme [39]. The strong inhibitory effect of all-trans astaxanthin on acetylcholinesterase was due to its unique chemical structure. One end of the long carbon chain of astaxanthin interacted with the catalytic site of acetylcholinesterase. The other end interacted with the peripheral anionic site. The intermediate carbon chain meets the distance requirement between the two sites and thus fit well with the entire acetylcholinesterase active site. In the astaxanthin-acetylcholinesterase complex structure, all-trans astaxanthin occupied with most of the space in the aromatic gorge. Thus, astaxanthin competed with substrate acetylthiocholine iodide then exhibited the potent inhibitory effect.

## 3. Conclusions

All-trans astaxanthin protects the cell viability of PC12 cells with a simultaneous inhibitory effect on acetylcholinesterase and oxidative stress. The natural carotenoid with multiple health benefits is a safe and efficient inhibitor. It can be a potential nutraceutical supplement to inhibit acetylcholinesterase.

## 4. Materials and Methods

### 4.1. Chemicals and Regents

Galantamine (purity > 98%) and peptide amyloid-β (Aβ25–35, purity > 97%) were purchased from Shanghai Yuanye Biotechnology Co., Ltd. (Shanghai, China). Acetylcholinesterase (from *Electrophorus electricus*, 500 units/mg), all-trans astaxanthin standard (purity > 97%), acetylthiocholine iodide (purity > 98%), 5′,5-dithiobis- (2-nitrobenzoic acid) (purity > 98%), analytical grade methanol, ethyl alcohol (purity > 95%), dimethyl sulfoxide, methyl tert-butyl ether, bovine serum albumin, penicillin (100 units/mL), and streptomycin (100 μg/mL) were purchased from Sigma-Aldrich Co (St. Louis, MO, USA). PC12 cell, 3-(4,5-dimethyl-2-thiazolyl)-2,5- diphenyl-2H-tetrazolium bromide (MTT), horse serum, and bicinchoninic acid protein assay kit were purchased from Solarbio Science &Technology Co., Ltd. (Beijing, China). RPMI 1640 medium was purchased from Fisher Scientific (Waltham, MA, USA). Fetal bovine serum was purchased from Gemini Bio-Products (West Sacramento, CA, USA). Superoxide dismutase, malondialdehyde, catalase, and acetylcholinesterase (Acetylcholinesterase) kits were purchased from Nanjing Jiancheng Bioengineering Institute (Nanjing, China).

### 4.2. In Vitro Assay of Acetylcholinesterase Inhibition

The in vitro inhibition of acetylcholinesterase activity was measured according to the reported method [40] with minor modifications. Firstly, 80 μL buffer D solution, 20 μL different final concentrations of sample solution (0, 1.63, 3.25, 6.50, 13.0, 26.0, and 52.0 μmol/L), and 20 μL 0.4 U/mL acetylcholinesterase solution were added in a 96-well microplate in order. Buffer D (pH = 8.0) was a mixture of 50 mmol/L Tris-HCl and 0.1% bovine serum albumin. After pre-warming at 37 °C for 15 min, 40 μL 0.6 mmol/L substrate acetylthiocholine iodide solution and 40 μL 0.6 mmol/L 5′,5-dithiobis- (2-nitrobenzoic acid) solution were added into the mixture and then incubated at 37 °C for another 15 min. Finally, 50 μL ethyl alcohol (greater than 95%) was added to stop the reaction. The absorbance of the mixture was recorded at 405 nm on a Unico^®^ 7200 spectrophotometer (Unico Instrument Corporation, Dayton, NJ, USA). The absorbance wavelength of all-trans astaxanthin (characteristic group) was at 480 nm. The sample blank group was prepared by adding 20 μL methanol instead of the sample solution. The control group was prepared by adding 20 μL inactivated acetylcholinesterase solution. The control blank group was prepared by adding 20 μL methanol instead of the sample solution and 20 μL inactivated acetylcholinesterase solution. The above control experiments were set to reduce the interference of residual astaxanthin in the mixture system at 405 nm. The positive control group was prepared by adding 20 μL galantamine solution instead of sample solution and other operations were same as described above. 

Acetylcholinesterase inhibitory rate (%) was calculated as follows:

(1) $$\mathrm{Inhibitory}\;\mathrm{rate}\;(\%)=\left[1-\frac{{\mathrm{OD}}_{\mathrm{sample}}-{\mathrm{OD}}_{\mathrm{sample}\;\mathrm{blank}}}{{\mathrm{OD}}_{\mathrm{control}}-{\mathrm{OD}}_{\mathrm{control}\;\mathrm{blank}}}\right]\times 100\%$$

where OD_sample_ is the absorbance of solution with the sample and active acetylcholinesterase; OD_sample blank_ is the absorbance of solution with methanol and activate acetylcholinesterase; OD_control_ is the absorbance of solution with the sample and inactivated acetylcholinesterase; OD_control blank_ is the absorbance of solution with methanol and inactivated acetylcholinesterase.

The IC_50_ value is defined as the sample concentration that is required to reach a 50% inhibition of acetylcholinesterase activity and measured by linear fitting.

### 4.3. Inhibition Kinetic Analysis

The method was referred to a previously reported protocol with minor modifications [24]. Briefly, 20 μL different final concentrations of all-trans astaxanthin solution (0, 13, 26, and 52 μmol/L) and 20 μL different concentrations of acetylcholinesterase solution (0.2, 0.4, 0.6, 0.8, 1.0 U/mL) were added in a 96-well microplate. After pre-warming at 37 °C for 15 min, 40 μL 0.6 mmol/L 5′,5-dithiobis-(2-nitrobenzoic acid) solution, and 40 μL 0.6 mmol/L substrate acetylthiocholine iodide solution were added into the mixture and then incubated at 37 °C for another 15 min. The reaction was stopped by adding 50 μL ethyl alcohol. Then, with the concentration of acetylcholinesterase solution as the x-axis and the reaction speed of the substrate as the y-axis, the reaction curve was drawn, and the change of slope was observed.

A total of 20 μL different final concentrations of all-trans astaxanthin solution (0, 6.5, 26, and 52 μmol/L) and 20 μL 0.4 U/mL acetylcholinesterase solution were added in a 96-well microplate and incubated at 37 °C for 15 min. After pre-warming at 37 °C for 15 min, 40 μL 0.6 mmol/L 5′,5-dithiobis-(2-nitrobenzoic acid) solution and 40 μL different final concentrations of substrate acetylthiocholine iodide solution (0.5, 1.0, 2.0, 4.0, 8.0 mmol/L) were added into the mixture and then incubated at 37 °C for another 15 min. The reaction was stopped by adding 50 μL ethyl alcohol. Then, with the reciprocal of the substrate concentration (1/[*S*]) as the *x*-axis and the reciprocal of (1/*V*) reaction speed as the *y*-axis, a double reciprocal curve was drawn. The inhibition type was determined by analyzing Lineweaver–Burk double reciprocal plots.

### 4.4. Fluorescence Spectroscopy Analysis

Fluorescence spectroscopy was used to study the conformation change of acetylcholinesterase when it was combined with different concentrations of all-trans astaxanthin. The method was referred to the protocol that was reported earlier [41]. A total of 925 μL buffer D solution, 50 μL 50 U/mL acetylcholinesterase solution and 25 μL astaxanthin solution (final concentration of 0, 50.0, 150, 250, and 350 μmol/L) were mixed and pre-warmed in a centrifuge tube at 37 °C for 15 min. Then, the mixture was transferred into a 1 cm-path length quartz cuvette.

The fluorescence quenching experiment was conducted via a Varian Cary Eclipse fluorescence spectrometer (Varian Inc., Palo Alto, CA, USA). The emission spectra were recorded at wavelengths of 310 to 450 nm. Other parameters were set as follows: the excitation wavelength was set at 280 nm, the excitation and emission slits were 5 nm, the scanning rate was 600 nm/min, and the resolution was 1.0 nm. All the data were obtained at 25 °C.

### 4.5. Circular Dichroism Spectroscopy Analysis

The circular dichroism spectroscopy experiment was conducted according to a reported method [41] with minor modifications. Firstly, 0.2 mL 50 U/mL acetylcholinesterase solution (in phosphate buffer, pH = 8.0) was mixed with 20 μL of all-trans astaxanthin solution (final concentration of 0, 50, and 100 μmol/L) in a centrifuge tube. Then, the mixture was transferred to a 1-cm-path length quartz cuvette. The intensity of the circular dichroism spectroscopy was recorded by using a Jasco-810 spectrophotometer (JASCO, Tokyo, Japan). The detection conditions were set as follows: the range of scan wavelength was 190 to 250 nm, the scanning rate was 100 nm/min, the resolution time was 0.5 s, the respond time was 1 s, and the bandwidth was 2 nm.

### 4.6. MTT Assay

MTT colorimetry was used to evaluate the effect of the all-trans astaxanthin concentration on PC12 cell viability according to a previously reported protocol [42]. Briefly, the cells were cultured to a density of 1 × 10^5^ cells/well at 37 °C for 24 h in 96-well microplate. Then, the supernatant was removed and 100 μL different final concentrations of astaxanthin (0, 8.37, 16.8, 33.6 μmol/L) was added in each microplate. After being incubated at 37 °C for another 24 h, the supernatant was removed. Then, 100 μL 5 mg/mL MTT solution (pH = 7.4) was added to each well, and incubated at 37 °C for 30 min. Then, the cells were gently washed twice with PBS buffer (pH = 7.4). A total of 100 μL dimethyl sulfoxide was added to lyse the cells, and the absorbance was measured at 570 nm. All the incubation was carried out at 37 °C, 5% CO_2_ and 95% relative humidity.

The cell viability was calculated according to following formula:

(2) $$\mathrm{Cell}\;\mathrm{viability}\left(\%\right)=\frac{\;{\mathrm{OD}}_{\mathrm{sample}\;570}}{\;{\mathrm{OD}}_{\mathrm{blank}\;570}}\times 100\%$$

where OD_sample 570_ is the absorbance of the solution with the sample (in different concentrations) at 570 nm. OD_blank 570_ is the absorbance of solution with dimethyl sulfoxide.

### 4.7. Cell Pretreatment

PC12 cells (concentration of 1× 10^5^ cells/mL) were cultured in a 96-well microplate at 37 °C in Dulbecco’s Modified Eagle’s Medium (Fisher Scientific, Waltham, MA, USA) in a humified atmosphere with 5% CO_2_ for 24 h. The growth of the PC12 cells was observed by an inverted microscope. The AD cell model was established as previously described [43] with minor modifications. Briefly, the cell supernatant was removed after incubating for 24 h with culture medium containing 100 μL methanol. Then, the cells were incubated for 24 h with 100 μL 20 μmol/L Aβ25–35 to establish the AD cell model. In astaxanthin-treatment group, 100 μL different concentration of astaxanthin solution (8.37, 16.8, 33.6 μmol/L) was added to cells and the mixture was incubated for 24 h. After removing the supernatant, the cells were cultured with 100 μL 20 μmol/L Aβ25–35 for 24 h. In the blank control group, 100 μL methanol was added to the cells as a blank control after 24 h of incubation and the cells was cultured for another 24 h.

### 4.8. Determination of Malondialdehyde Content and the Activities of Acetylcholinesterase, Superoxide Dismutase, and Catalase

The PC12 cells of each group were collected and subjected to cell lysis. After centrifugation at 14,000 g for 10 min, the supernatant was collected. The protein content in each supernatant sample was determined using the bicinchoninic acid protein assay kit (Transgen, Beijing, China) and adjusted to be consistent. The ELISA method was used to detect the changes of the oxidative damage index for superoxide dismutase and catalase according to the kit instructions. Acetylcholinesterase and malondialdehyde in the supernatant were also measured with corresponding kits according to the instructions.

### 4.9. Molecular Docking Analysis

The X-ray co-crystal structure of Acetylcholinesterase (PDB code: 1EVE) was obtained from the Protein Data Bank (http://www.rcsb.org, data accessed on 10 September 2021). The 2D structures of all-trans astaxanthin, galantamine, and substrate acetylthiocholine iodide were acquired from the PubChem database (https://pubchem.ncbi.nlm.nih.gov/, data accessed on 9 September 2021). Discovery Studio 2.5 software (Accelrys Inc., San Diego, CA, USA) was used to perform the molecular docking studies. Autodock 4.2 molecular docking software (The Scripps Research Institute, San Diego, CA, USA) was used to determine the binding energies. The docked complex conformations were analyzed by hydrogen bonding, hydrophobicity, electrostatics, entropy, and C-π interaction.

### 4.10. Statistical Analysis

All the tests were performed in triplicate and the results were expressed as means ± standard deviation (SD). The data were analyzed by one-way analysis of variance (ANOVA) and statistical significance was defined as *p* < 0.05. All the analyses were done by using Microsoft Office Excel 2016, Origin 8.0 and SPSS 17.0.

## Data Availability

Not applicable.
